# Virus-to-prokaryote ratio in the Salar de Huasco and different ecosystems of the Southern hemisphere and its relationship with physicochemical and biological parameters

**DOI:** 10.3389/fmicb.2022.938066

**Published:** 2022-08-18

**Authors:** Yoanna Eissler, Alonso Castillo-Reyes, Cristina Dorador, Marcela Cornejo-D'Ottone, Paula S. M. Celis-Plá, Polette Aguilar, Verónica Molina

**Affiliations:** ^1^Instituto de Química y Bioquímica, Facultad de Ciencias, Universidad de Valparaíso, Valparaíso, Chile; ^2^Escuela de Biología Marina, Facultad de Ciencias del Mar y de Recursos Naturales, Universidad de Valparaíso, Viña del Mar, Chile; ^3^Laboratorio de Complejidad Microbiana y Ecología Funcional, Instituto de Antofagasta, Departamento de Biotecnología, Facultad de Ciencias del Mar y Recursos Biológicos, Universidad de Antofagasta, Antofagasta, Chile; ^4^Centre for Biotechnology and Bioengineering, Universidad de Chile, Santiago, Chile; ^5^Escuela de Ciencias del Mar e Instituto Milenio de Oceanografía, Pontificia Universidad Católica de Valparaíso, Valparaíso, Chile; ^6^Laboratory of Aquatic Environmental Research, Centro de Estudios Avanzados, Universidad de Playa Ancha, Viña del Mar, Chile; ^7^HUB Ambiental UPLA, Universidad de Playa Ancha, Valparaíso, Chile; ^8^Departamento de Ciencias y Geografía, Facultad de Ciencias Naturales y Exactas, Universidad de Playa Ancha, Valparaíso, Chile; ^9^Centro de Investigación Oceanográfica COPAS COASTAL, Universidad de Concepción, Concepción, Chile

**Keywords:** virus, bacteria, prokaryote, relationships, aquatic ecosystems, VBR, high-altitude wetland, VPR

## Abstract

The virus-to-prokaryote ratio (VPR) has been used in many ecosystems to study the relationship between viruses and their hosts. While high VPR values indicate a high rate of prokaryotes' cell lysis, low values are interpreted as a decrease in or absence of viral activity. Salar de Huasco is a high-altitude wetland characterized by a rich microbial diversity associated with aquatic sites like springs, ponds, streams and a lagoon with variable physicochemical conditions. Samples from two ponds, Poza Rosada (PR) and Poza Verde (PV), were analyzed by epifluorescence microscopy to determine variability of viral and prokaryotic abundance and to calculate the VPR in a dry season. In addition, to put Salar de Huasco results into perspective, a compilation of research articles on viral and prokaryotic abundance, VPR, and metadata from various Southern hemisphere ecosystems was revised. The ecosystems were grouped into six categories: high-altitude wetlands, Pacific, Atlantic, Indian, and Southern Oceans and Antarctic lakes. Salar de Huasco ponds recorded similar VPR values (an average of 7.4 and 1.7 at PR and PV, respectively), ranging from 3.22 to 15.99 in PR. The VPR variability was associated with VA and chlorophyll *a*, when considering all data available for this ecosystem. In general, high-altitude wetlands recorded the highest VPR average (53.22 ± 95.09), followed by the Oceans, Southern (21.91 ± 25.72), Atlantic (19.57 ± 15.77) and Indian (13.43 ± 16.12), then Antarctic lakes (11.37 ± 15.82) and the Pacific Ocean (6.34 ± 3.79). Physicochemical variables, i.e., temperature, conductivity, nutrients (nitrate, ammonium, and phosphate) and chlorophyll *a* as a biological variable, were found to drive the VPR in the ecosystems analyzed. Thus, the viral activity in the Wetland followed similar trends of previous reports based on larger sets of metadata analyses. In total, this study highlights the importance of including viruses as a biological variable to study microbial temporal dynamics in wetlands considering their crucial role in the carbon budgets of these understudied ecosystems in the southern hemisphere.

## Introduction

Viruses are the most abundant components in the biosphere, far exceeding the number of bacteria and, anecdotally, even exceeding the number of stars in the visible universe (Griffin, 2013). Only in the oceans virus-like particles (VLP) reach between 10^4^ to 10^8^ per milliliter of seawater (Weinbauer, 2004; Clasen et al., 2008a; Payet et al., 2014) and 10^5^ to 10^7^ VLP mL^−1^ in others aquatic ecosystems such as wetlands (Jackson and Jackson, 2008). Viruses are dynamic and important components in many terrestrial and marine ecosystems (Srinivasiah et al., 2008). They can control the composition of communities, influence biogeochemical cycles, and induce horizontal gene transfer (Syvanen, 2012; Breitbart et al., 2018).

The virus to prokaryotic ratio (VPR) has been widely used by researchers (Wommack and Colwell, 2000; Weinbauer, 2004; Parikka et al., 2016) to study the relationship between viral communities and other microorganisms (whether bacteria or archaea) in the environment. This index determines the incidence that viruses have on their hosts, i.e., high values of VPR indicate that a high viral dynamic is taking place and, consequently, a high rate of prokaryotic cell lysis (Wommack and Colwell, 2000). In contrast, low values are interpreted as a decrease in or absence of viral activity (Wommack and Colwell, 2000; Weinbauer, 2004; Parikka et al., 2016). Despite the differences in the amount of VLPs in environments such as oceans, rivers, lakes, groundwater, soil, and marine sediments (Clasen et al., 2008b; Srinivasiah et al., 2008; Parikka et al., 2016), in most ecosystems the abundance of viruses far exceeds that of their prokaryotic hosts by at least an order of magnitude (Breitbart and Rohwer, 2005; Wigington et al., 2016). This can also be observed in the VPR averages of different ecosystems, being 20.7 in coastal marine environments, 38 in the open ocean, 11.3 in estuaries, and 18.8 in lakes (Parikka et al., 2016). These differences in VPR values may be caused by diverse biological (e.g., chlorophyll *a* concentration, and abundance of bacteria) (Clasen et al., 2008b) and physicochemical factors (e.g., temperature, salinity, and nutrients) (Tuomi et al., 1995; Eissler et al., 2010; Salter et al., 2011; Parikka et al., 2016). In eutrophic environments, viral production (VP), i.e. the number of viruses produced in a given volume and time span, is associated with prokaryotic production, which eventually reduces prokaryotes abundance due to viral lysis (Steward et al., 1996; Noble and Fuhrman, 2000; Wilhelm et al., 2002; Winget and Wommack, 2009). These top-down controls of virus activity on prokaryotes consequently result in an increase in virus abundance and VPR values (Wommack and Colwell, 2000; Parikka et al., 2016). In contrast, there is less prokaryotic production in oligotrophic environments, so viral production and VPR values are low (Parikka et al., 2016). In particular, the southern hemisphere concentrates conspicuous microbial diversity hotspots systems associated with trophic gradients, like the eutrophic coastal areas of the Eastern South Pacific an Eastern Upwelling Boundary System (EBUS) with prominent oxygen minimum zones (OMZ) and Anoxic Marine Zones in the waters (Ulloa et al., 2012). In this EBUS area, VPR values have been shown to decrease from the surface and then increase again in the sub-oxic or anoxic subsurface layer (Taylor et al., 2003; Chiang and Quiñones, 2007). High VPR values have been registered in coastal bay areas at EBUS (Kuznar et al., 2009; Eissler et al., 2010).

On the other hand, the southern hemisphere concentrates a significant number of extreme and poly extreme ecosystems understudied in terms of VPR dynamics, such as the highlands Andean wetlands. High-altitude wetlands, such as Salar de Huasco, have been considered a polyextreme environment, i.e., where multiple and/or simultaneous forms of stress determine diversity (Dorador et al., 2020). This ecosystem is located at 3,800 m above sea level at the Chilean Altiplano and covers an area of approximately 15,858 ha, with the presence of permanent brackish lagoons, ephemeral hypersaline ponds, and freshwater springs (Dorador et al., 2020). Regarding viral activity, a wide range of VPR values, from 2 to 351 (Eissler et al., 2019), have been found in this wetland. VPR values varied greatly associated with the high heterogeneity of aquatic environments within the Salar de Huasco, especially considering salinity ranging from fresh to mesosaline water (Eissler et al., 2019, 2020).

In this study, we aim to determine viral host dynamic relationships using VPR under different physical and chemical conditions in Salar de Huasco. Moreover, our results were compared with VPR variability in ecosystems characterized by contrasting origin and biogeochemical conditions from the southern hemisphere.

## Materials and methods

### Sampling sites

**Figure 1:**
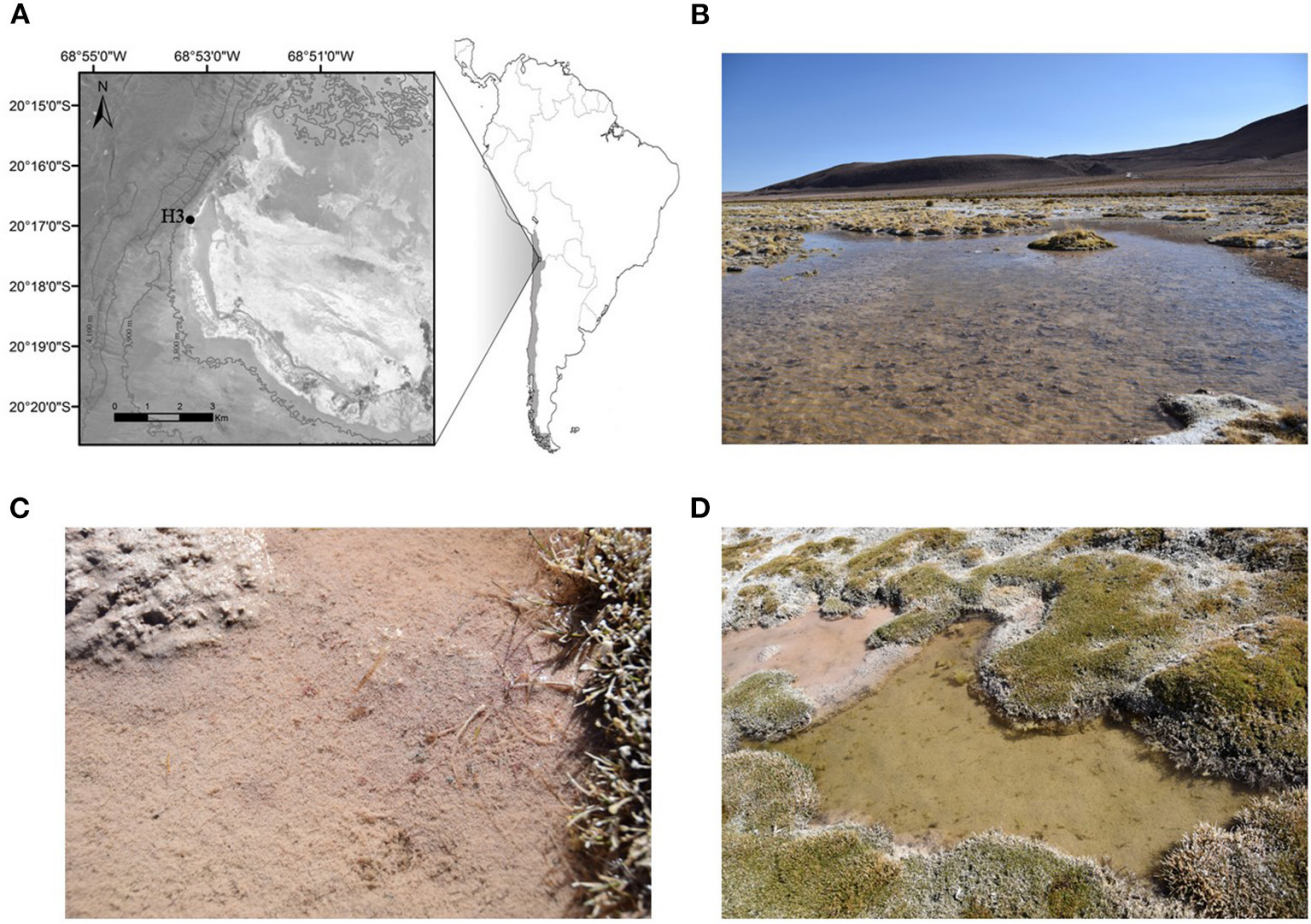
**(A)** Map showing the location and area of the site (H3) sampled during 16-17 September 2019, **(B)** Shows a general imagine of “Poza Rosada” (PR), **(C)** Shows a close up of microbial mats that are characterized by their pink color and **(D)** Shows “Poza Verde” (PV) and its green color in contrast with a near pond of pink color.

The sampling was carried out during the dry season in Salar de Huasco, Chile, (September 16-17, 2019). Samples were taken from two ponds located at site H3 (20° 17'01”S; 68° 53'21”W) (Figure 1A), referred to as “Poza Rosada” (PR) and “Poza Verde” (PV) due to their qualitative characteristics in relation to their respective predominant colors (Figures 1B–D). PR was sampled five times during 2 days and PV was sampled twice for 1 day.

### Measurement of physicochemical and biological variables

The physical and chemical parameters of these sites (i.e., temperature, conductivity and pH) were measured *in situ* with a Thermo Scientific Orion Star multiparameter device (model A329). To determine nitrate, nitrite, phosphate and silicic acid, water samples (60 mL) were taken in triplicate, filtered through a GF/F filter (Whatman^®^, Buckinghamshire, UK) and stored at −20°C. These nutrient samples were analyzed using colorimetric methods (Strickland and Parsons, 1972) with an automatic nutrient analyzer (Atlas et al., 1971). To determine chlorophyll-a concentration, 200 mL water samples were filtered through a GF/F filter (Whatman^®^, Buckinghamshire, UK). The filters were stored at −20°C to then be analyzed using the fluorometric method (Caspers, 1970) in a Turner A10 fluorometer.

### Viruses and prokaryotes abundance

Samples were taken with 15 mL centrifuge tubes, fixed with 1% glutaraldehyde and immediately frozen until analysis. To count virioplankton, the viral samples were pre-filtered using a syringe and a 0.22 μm polyethersulfone filter unit (Millex GP, Millipore^®^, Darmstadt, Germany), followed by filtration through an Anodisc membrane filter (Whatman^®^, Buckinghamshire, UK) of 0.02 μm pore size, applying 130 mm Hg of pressure. Each filter was placed in a Petri dish in the dark and stained for 15 min with 100 μl of SYBR^TM^-Gold at a concentration of 2x (10000X stock solution; Invitrogen, Carlsbad, CA, USA), which was prepared from a 50x intermediate solution with molecular biology grade water previously filtered by 0.025 μm MF^TM^ membrane filters (Millipore^®^, Darmstadt, Germany). Once the filters were stained, the excess solution was removed with Kimwipe^®^ tissues (Kimtechscience, Little Rock, AR, USA) and they were allowed to dry for approximately 10 min on a filter paper in the dark. Finally, each filter was mounted on a slide for later observation. To estimate the abundance of prokaryotes, the same procedures described for the viral samples were conducted, omitting the pre-filtering of the sample, and using an Anodisc membrane filter (Whatman^®^, Buckinghamshire, UK) of 0.2 μm pore size to mount the samples (Chen et al., 2001). VLPs and prokaryotes were observed at 1000x magnification through an epifluorescence microscope (Olympus BX60F-3, equipped with a WIB long-pass fluorescence cube, excitation wavelength 460-490 nm, DM 505 beam splitter, emission BA 515IF) fitted with a 50 W HBO mercury lamp, where a total of at least 100 viral particles and cells were counted in 10 to 20 randomly chosen fields.

### Data processing

Literature review of articles on microbial communities of the Salar de Huasco and other aquatic ecosystems of the Southern hemisphere was carried out. Research articles with the original viral abundance and prokaryote data available in tables, either in results or Supplementary Files, were selected. Articles with graphical representation of results had to be excluded. The articles were obtained from different databases (ScienceDirect, SciELO, PubMed and Google Scholar). The search was carried out by entering the following keywords: “virus-to-prokaryote ratio”, “virus-to-bacteria ratio”, “virus-to-cell ratio”, “Virus-to-host ratio”, “VPR”, “VBR”, “Salar de Huasco”, “viral abundance” and “viral activity”, applying the same criteria used by Parikka et al. (2016). Two of the articles found did not present the data in a tabular format, for which reason the authors were contacted for data details (Chiang and Quiñones, 2007 and Eissler et al., 2010). When VPR was not available in the article, viral and prokaryotic abundances were used to calculate this index. Units had to be converted, when necessary, to enable statistical analyses. In the case of salinity, practical salinity units and parts-per-thousand (‰) were considered as equivalent. Salinity was transformed to conductivity using the salinity conversion calculator available at: https://www.hamzasreef.com/Contents/Calculators/SalinityConversion.php, following Wagner et al. (2006). These data were classified on the basis of habitat (e.g., ponds, springs, marine, freshwater) and ecosystems type (e.g., high-altitude wetlands, oceans, and lakes) and are available, along with the physicochemical and biological variables, in Supplementary Table S1.

### Data and statistical analysis

First, Shapiro-Wilk, Kolmogorov-Smirnov and Lilliefors tests were conducted to check the normality of all data. Data distribution was not normal (Supplementary Table S2). Therefore, statistical analysis to compare VPR values of the different ecosystems were carried out with a non-parametric test like Kruskal Wallis and Tukey test (Statistica version 7.0). To determine relationships between the VPR values and the biological and environmental parameters of the Salar de Huasco and all ecosystems, a non-parametric Spearman's rank correlation was performed (Statistica version 7.0).

VPR distribution of the different ecosystems analyzed, considering the physicochemical conditions such as temperature, conductivity and depth, were visualized using bubble plots with ggplot2 and geom_jitter in R studio (R Core Team, 2021).

Principal component analysis (PCA) was performed with the environmental data normalized by using scale function in R studio, to visualize the physicochemical variability in different habitats of Salar de Huasco, i.e., ponds, springs, and lagoon (database *n* = 16) and ecosystems, i.e., high-altitude wetlands, Pacific Ocean, Atlantic Ocean, and Southern Ocean (database *n* = 74), then the envfit function (library vegan) was used to overlay viral, prokaryotic abundances and VPR and determine their potential association with the ordination axes (R Core Team, 2021). SigmaPlot version 14.0 program was used to plot the variation of viral and prokaryotic abundances and other environmental variables studied from PV and PR in Salar de Huasco, and was also used to compare VPR values of the studied ecosystems.

## Results

### Data

**Figure 2:**
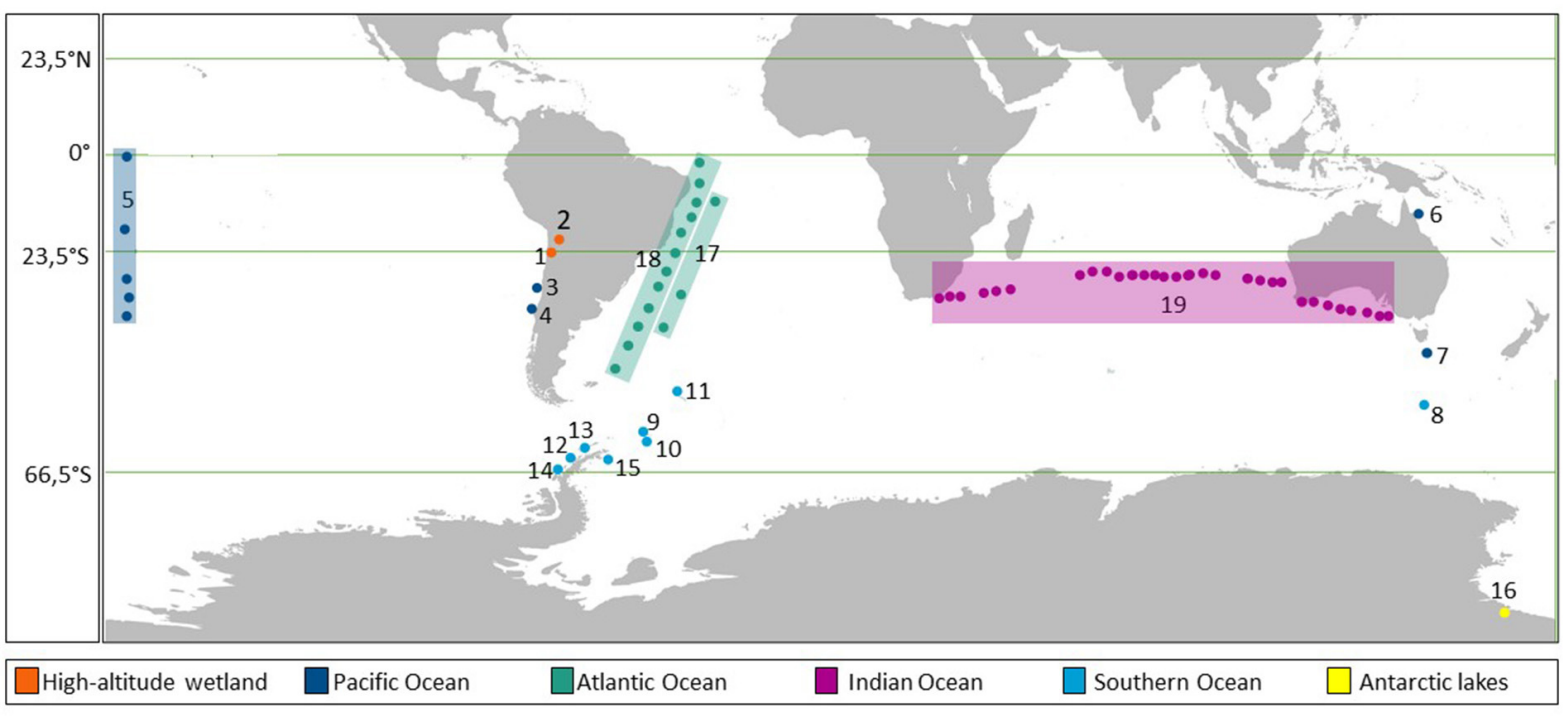
Map showing the areas and sampling sites analyzed in this work from the Southern hemisphere. The numbers (ID) show the areas sampled and colored squares indicate sampled transects.

A total of 15 reports on microbial abundances, including viruses and environmental parameters from 19 areas of different ecosystems in the Southern hemisphere, were considered for comparison (Figure 2; Table 1). Together, these publications account for a total of 1198 viral and prokaryotic abundance data with their respective VPR (Supplementary Table S1). The following physical, chemical and biological conditions, i.e., dissolved oxygen, pH, salinity, concentrations of nitrate, nitrite, ammonium, phosphate, silicate, chlorophyll, carbon dioxide, methane, nitrous oxide, when available (Supplementary Table S1), were listed. The areas were divided on the basis of the large ecosystems of origin (i.e., high-altitude wetlands, Pacific, Atlantic, Indian and Southern Oceans and Antarctic Lakes, Figure 2).

**Table 1:** Mean values of viral (VA) and prokaryotic abundance (PA); average, standard deviation (SD) and range (minimum to maximum) of VPR in aquatic ecosystems of South hemisphere.

**Location**	**Area ID**	**Coordinates**	**VA**	**PA**	**VPR**		**Reference**
			**VLP mL^−1^**	**Cells mL^−1^**	**Average (SD)**	**Min–max**	
Salar de Huasco	1	20°17′01”S 68°53′21”W	1.20 × 10^8^	4.78 × 10^6^	56.68 ± 106.01	1.15–351	(This work; Molina et al., 2018; Eissler et al., 2019, 2020)
Salar de Uyuni	2	20°08′01.6”S 7°29'20.9”W	1.15 × 10^8^	1.94 × 10^7^	42.33 ± 51.78	0.51–122.5	Ramos-Barbero et al., 2019
Bajo O'Higgins Seamount, Valparaíso	3	32°54′S 73°53′W	5.51 × 10^9^	6.52 × 10^8^	7.65 ± 3.10	5.63–9.64	Chiang and Quiñones, 2007
Coastal upwelling system, Concepción	4	36°29.94′S 73°07.8′W	1.84 × 10^7^	2.12 × 10^6^	7.80 ± 9.31	0.25–45.73	Eissler et al., 2010
Thermohaline circulation, South Pacific	5	40°00'45.0“S 9°59'43.8”W 0°00'12.0”N 69°59'53.9”W	1.33 × 10^5^	8.06 × 10^3^	16.62 ± 2.42	12.76–18.36	De Corte et al., 2019
Great Barrier Reef, Australia	6	15.55405°S 145.45964°E 19°16'33.7”S 47°03'26.8”E	7.07 × 10^6^	1.31 × 10^6^	5.29 ± 1.40	2.82–8.45	Carreira et al., 2020
Sub-Antarctic Zone	7	50.003°S 149.442°E 45.441°S 153.290°E	1.40 × 10^10^	1.94 × 10^9^	8.35 ± 2.74	5.25–13.87	Evans et al., 2009
Polar Frontal Zone	8	54.140°S 146.296°E 54.002°S 145.878°E	6.63 × 10^9^	6.90 × 10^8^	9.64 ± 0.92	8.70–10.75	Evans et al., 2009
North of South Orkney	9	60°00'20.2“S 5°31'35.4”W	3.82 × 10^6^	2.31 × 10^5^	17.48 ± 14.22	1.52–60.98	Sotomayor-Garcia et al., 2020
South of South Orkney	10	61°09'11.2“S 43°30'51.5”W	3.18 × 10^6^	3.29 × 10^5^	10.62 ± 6.23	3.90–27.09	Sotomayor-Garcia et al., 2020
North of South Georgia	11	50°09'45.0”S 9°29'41.6”W	1.92 × 10^7^	5.35 × 10^5^	38.08 ± 21.87	15.94–87.50	Sotomayor-Garcia et al., 2020
West of Anvers	12	64°17'44.2”S 64°30'16.6”W	1.28 × 10^7^	3.40 × 10^5^	40.56 ± 18.31	12.31–68.18	Sotomayor-Garcia et al., 2020
Bransfield Strait	13	61°35'45.2“S 55°24'19.4”W 63°20'08.9”S 61°09'38.2”W	5.82 × 10^6^	4.43 × 10^5^	17.15 ± 17.67	1.09–89.50	Vaqué et al., 2017
Bellinghausen Sea	14	63°24'00.0”S 62°32'45.2”W 69°01'24.6”S 75°03'41.4”W	1.17 × 10^7^	5.83 × 10^5^	28.52 ± 41.95	1.00–208.97	Vaqué et al., 2017
Weddell Sea	15	61°24'20.5“S 52°18'00.4”W 65°00'39.6”S 55°27'09.4”W	4.12 × 10^6^	3.08 × 10^5^	15.01 ± 16.14	0.53 - 65	Vaqué et al., 2017
McMurdo Dry Valleys, Antarctica	16	77°37'27.9“S 162°59'41.5”E	1.04 × 10^7^	6.23 × 10^5^	11.37 ± 11.55	0.60 - 53	Lisle and Priscu, 2004
Thermohaline circulation, South Atlantic	17	0°11'29.4“S 32°52'28.2”W 39°57'53.3'S 42°25'23.5”W	5.93 × 10^5^	2.17 × 10^4^	29.01 ± 11.55	12.67–40.16	De Corte et al., 2019
Atlantic Ocean	18	0°11'29.4”S 32°52'28.2”W 49°32'51.0”S 52°41'18.6”W	1.96 × 10^6^	1.67 × 10^5^	19.35 ± 15.80	0.92–87.93	Alves Junior et al., 2015; De Corte et al., 2016
Indian Ocean	19	34°02'41.3”S 25°42'44.7”E 36°29'31.0”S 138°10'58.4”E	4.64 × 10^6^	4.37 × 10^5^	13.43 ± 16.12	0.61–127.6	Lara et al., 2017

### Viral abundance, VPR and environmental variables in the Salar de Huasco

**Figure 3:**
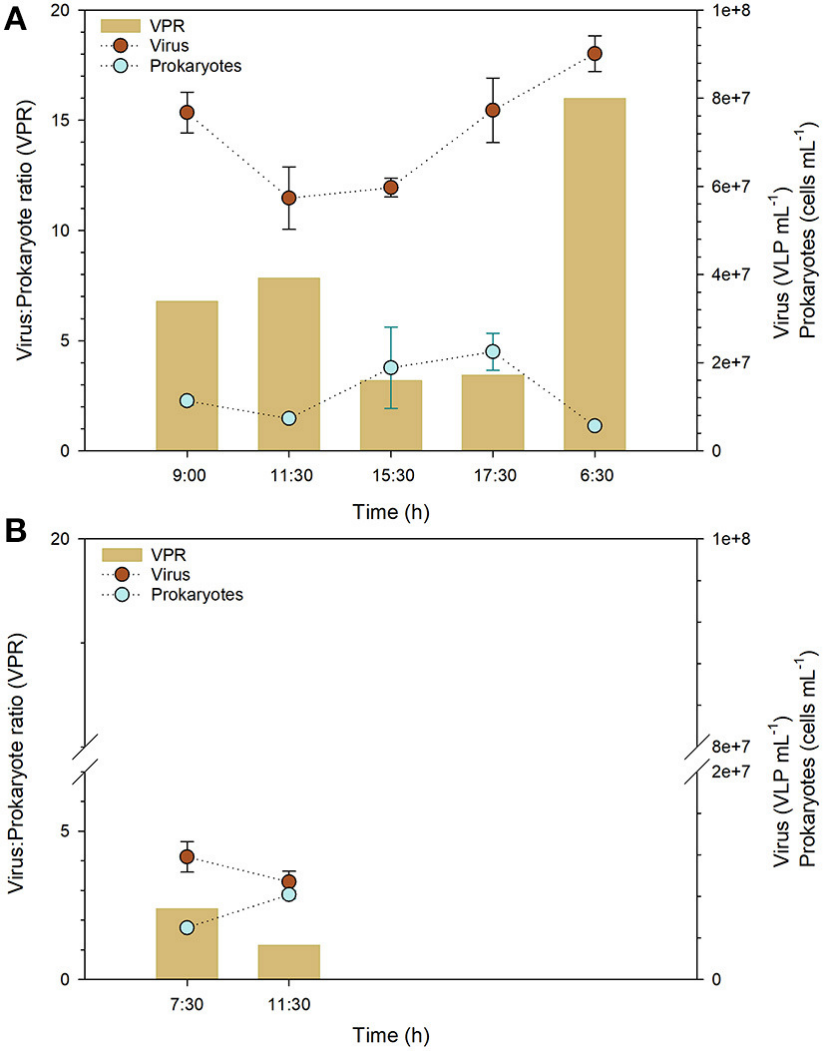
Viral and prokaryotic abundance and virus to prokaryote ratios (VPR) in two ponds sampled in Salar de Huasco (September 16-17th, 2019), **(A)** PR and **(B)** PV. Error bars represent the standard deviation.

During the daily cycle studied in PR, an increase in viral abundance was observed in the evening. A minimum value (5.73 × 10^7^ ± 7.08 × 10^6^ VLP mL^−1^) was observed around midday, at 11:30 h, while a maximum value of 9.01 × 10^7^ ± 4.06 × 10^6^ VLP mL^−1^ was registered at 6:30 h the next day, (Figure 3A; Supplementary Table S3). Prokaryotic abundance peaked in the afternoon (2.25 × 10^7^ ± 4.22 × 10^6^ cells mL^−1^), at 17:30 h, and reached its lowest value (5.64 × 10^6^ ± 3.54 × 10^5^ cells mL^−1^) at 6:30 h, which is consistent with the time at which the maximum viral abundance was found (Figure 3A; Supplementary Table S3). The VPR values during the daily cycle ranged from 3.17 to 15.99, with the minimum being observed at 15:30 h and the maximum at 6:30 h of the following sampling day (Figure 3A; Supplementary Table S3). Similar to PR, the viral abundance for PV was higher in the morning than at midday (1.18 × 10^7^ ± 1.46 × 10^6^ VLP mL^−1^, 7:30 h, 9.39 × 10^6^ ± 1.03 × 10^6^ VLP mL${}_{,}^{-1}$ 11:30 h) (Figure 3B; Supplementary Table S3). In contrast, prokaryotic abundance was lower at noon (Figure 3B), thus, the higher VPR value (2.38) was registered at 7:30 h (Figure 3B; Supplementary Table S3).

**Figure 4:**
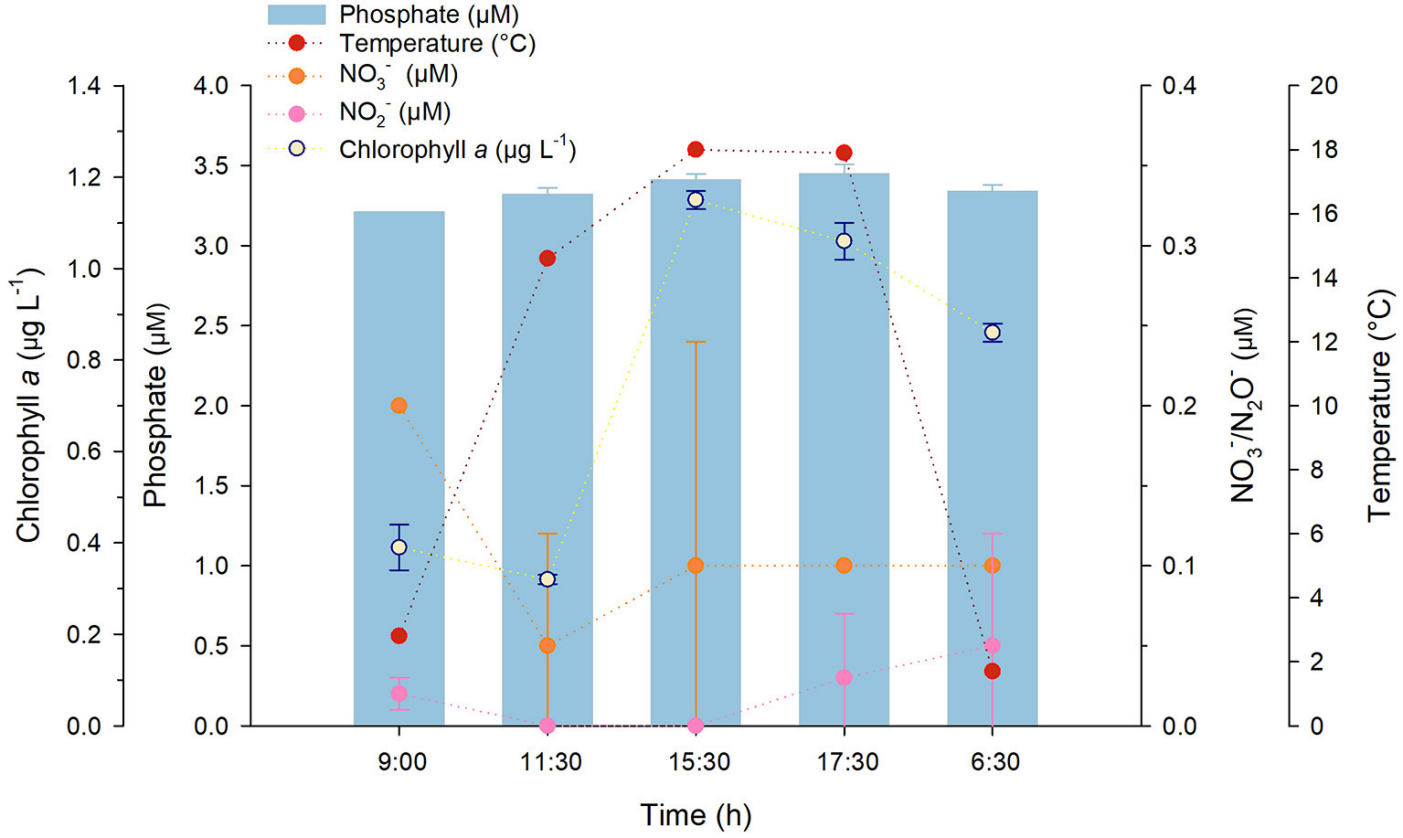
Temperature, nitrate, nitrite phosphate and chlorophyll *a* concentration changes in PR during sampling in Salar de Huasco (September 16–17th, 2019). Error bars represent the standard deviation.

Generally, there was not a significant nutrient variation over time; however, a variation in temperature and chlorophyll *a* concentrations, which were inversely related to viral abundance and VPR (Figures 3, 4), was recorded at PR. Regardless of the little data available, when relationships were examined, temperature presented no significant correlations with any of the variables, and chlorophyll *a* only showed a significant relation (*r* = −0.89) with conductivity (Supplementary Table S4). Viruses showed a significant correlation with Nitrite (*r* = 0.767) and Silicic acid (*r* = 0.821) and VPR with conductivity, pH (both *r* = 0.9) and chlorophyll *a* (*r* = 0.893); conversely, no relationships were found for prokaryotic abundance (Supplementary Table S4).

**Figure 5:**
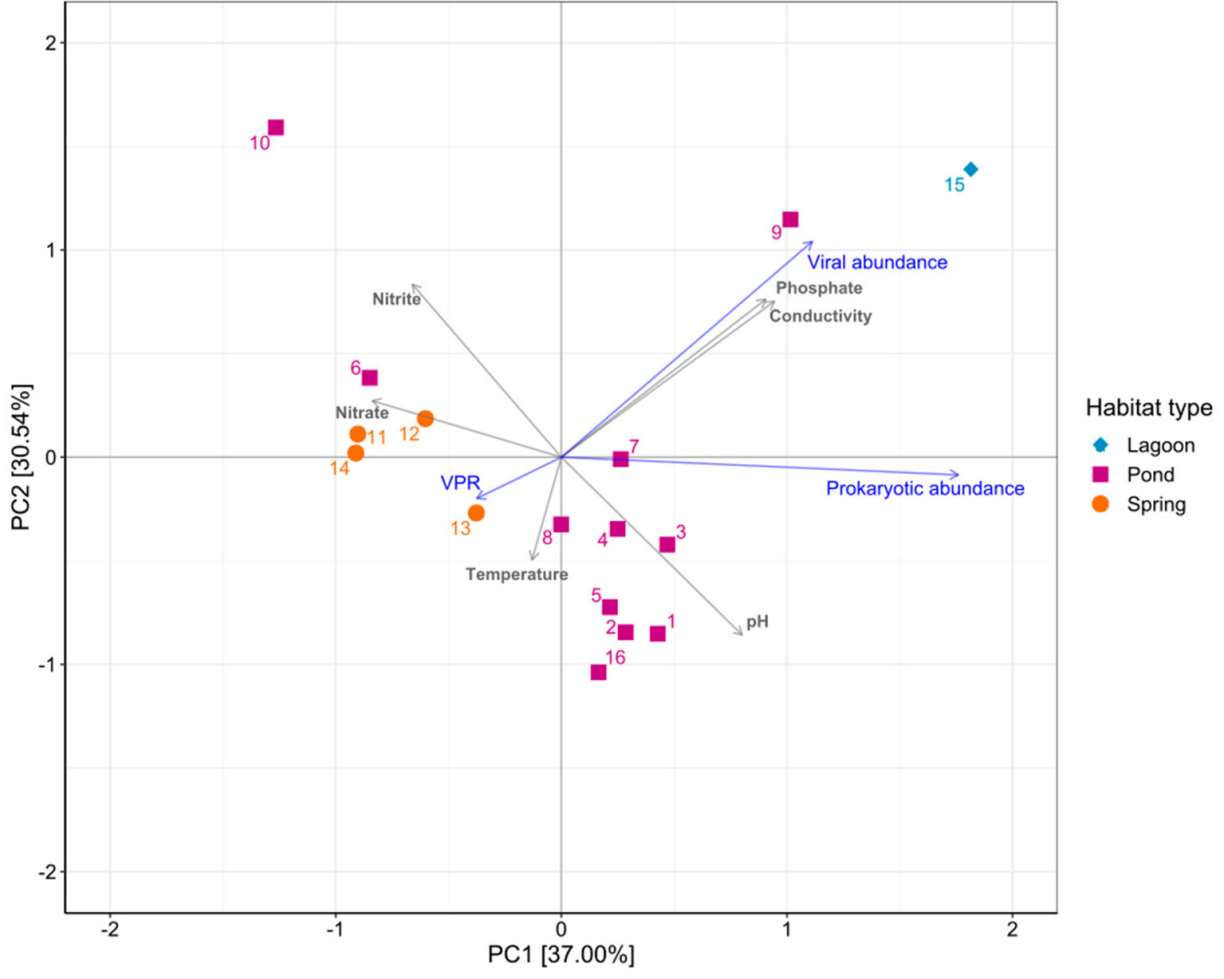
Principal component analysis showing the variability of environmental variables and VPR values associated with the different habitats studied in Salar de Huasco (data from this study, Molina et al., 2018; Eissler et al., 2019, 2020).

When all data available up to now for Salar de Huasco was correlated, significant relationships were found between viral abundance and prokaryotic abundance, VPR, conductivity, pH and Silicic acid (Supplementary Table S5). Prokaryotic abundance was significantly correlated with conductivity and nitrate, and VPR was significantly correlated with VA and chlorophyll *a* concentration Supplementary Table S5). On the other hand, the PCA ordination axes accounted for > 67%, of the variability associated with the environmental conditions of the different aquatic areas of Salar de Huasco, i.e, ponds, springs and lagoons as categories (Figure 5). Ponds were the habitat exhibiting a higher variability related with conductivity, pH and phosphate. Viral and prokaryotic abundance were positively correlated (*r* = 0.73 and 0.998, *P* ≥ 0.07), whereas VPR was negatively correlated (*r* = −0.88, *P* = 0.92) to PC1, axis accounting 37% of the aquatic environments' variability.

### VPR in different ecosystems of the southern hemisphere

A wide range of VPR values were found among all the ecosystems studied, varying from 0.25 in coastal upwelling system, Concepción to a maximum value of 351 in Salar de Huasco (Table 1), showing a difference of more than three orders of magnitude.

**Figure 6:**
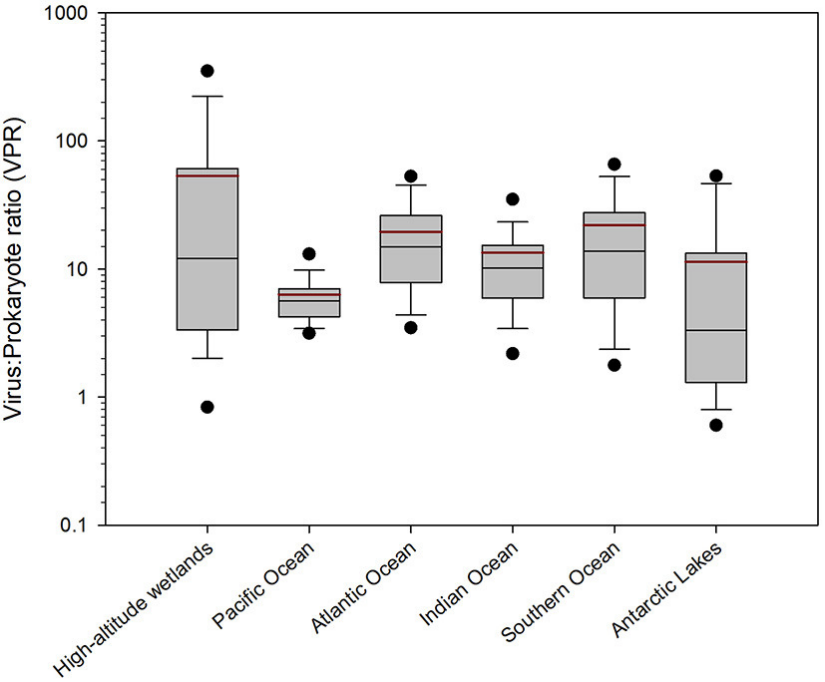
Boxplot graph of the VPR values of different aquatic ecosystems in the Southern hemisphere. The area of the boxes shows the 25th and 75th percentiles and the whiskers represent 5 and 95% of the data. The thin black line is the median and the thick dark red line is the average. Outliers are shown in black circles.

The high-altitude wetlands (i.e., Salar de Huasco and Salar de Uyuni) recorded the highest average VPR value (53.22 ± 95.09), followed by the Southern (21.91 ± 25.72), Atlantic (19.57 ± 15.77) and Indian (13.43 ± 16.12) Oceans (Figure 6). On the other hand, the Antarctic lakes and the Pacific Ocean recorded the lowest average VPR values, being 11.37 ± 15.82 and 6.34 ± 3.79, respectively (Figure 6).

The Kruskal-Wallis H test showed significant differences among the ecosystems' VPR values (*H* = 241.8; *P* < 0.001). Particularly, a Tukey test comparison showed that VPR values for the Pacific Ocean were significantly different from all the other ecosystems (*P* < 0.05), except for the Antarctic lakes (Table 2). The high-altitude wetlands showed significant differences with the Pacific Ocean and Antarctic lakes (*P* < 0.05), while the Atlantic Ocean showed significant differences with the Indian Ocean and Antarctic lakes (*P* < 0.05) (Table 2). Finally, the Indian Ocean and the Antarctic lakes showed significant differences with the Southern Ocean (*P* < 0.05) (Table 2).

**Table 2:** Tukey test comparison of VPR values of the South hemisphere ecosystems.

	**Pacific Ocean**	**Atlantic Ocean**	**Indian Ocean**	**Southern Ocean**	**Antarctic Lakes**
High-altitude wetlands	**<0.001**	0.672	0.935	0.992	**0.039**
Pacific Ocean	-	**<0.001**	**<0.001**	**<0.001**	0.999
Atlantic Ocean		-	**<0.001**	0.303	**<0.001**
Indian Ocean			-	**0.019**	0.054
Southern Ocean				-	**<0.001**

Significant differences (P < 0.05) are marked in bold.

Significant and positive correlations using the Spearman Rank test were found for VPR values and viral abundance, nitrate, ammonium, phosphate, and chlorophyll *a* (*P* < 0.05) (Table 3). In addition, VPR values presented a significant and negative correlation with prokaryotic abundance, temperature, and conductivity (*P* < 0.05) (Table 3). While viral abundance of the ecosystems was positive and significantly correlated with the abundance of prokaryotes, VPR, temperature, nitrate, and phosphate, it was negatively correlated with silicic acid and chlorophyll *a* (*P* < 0.05; Table 3). Finally, prokaryotic abundance was significant and positively correlated with viral abundance, temperature and conductivity and negatively related to VPR values, nitrate, ammonium, silicic acid, and chlorophyll *a* (*P* < 0.05) (Table 3).

**Table 3:** Spearman's rank correlations (r) between viral and prokaryotic abundance, VPR and environmental variables in all studied ecosystems of South hemisphere.

	**VPR**		**VA**		**PA**		
	***r***	***P***	***r***	***P***	***r***	***P***	***N***
VA (VLP mL^−1^)	**0.107**	**0.000**			**0.801**	**0.000**	1177
PA (cells mL^−1^)	**−0.450**	**0.000**	**0.801**	**0.000**			1177
VPR			**0.107**	**0.000**	**−0.450**	**0.000**	1177
Temperature (°C)	**−0.297**	**0.000**	**0.342**	**0.000**	**0.512**	**0.000**	1035
Conductivity (μS cm^−1^)	**−0.220**	**0.000**	−0.040	0.306	**0.214**	**0.000**	665
Dissolved oxygen (mg L^−1^)	0.183	0.064	−0.168	0.094	−0.171	0.087	101
pH	−0.004	0.984	−0.020	0.924	−0.077	0.719	24
NO${}_{3}^{-}$ (μM)	**0.538**	**0.000**	**0.217**	**0.000**	**−0.150**	**0.007**	321
NO${}_{2}^{-}$ (μM)	0.102	0.321	0.074	0.471	0.117	0.255	95
NH${}_{4}^{+}$ (μM)	**0.351**	**0.000**	0.035	0.732	**−0.370**	**0.000**	96
Silicic acid (μM)	0.109	0.267	**−0.428**	**0.000**	**−0.529**	**0.000**	105
Phosphate (μM)	**0.501**	**0.000**	**0.230**	**0.000**	−0.045	0.435	301
Chlorophyll *a* (μg L^−1^)	**0.320**	**0.000**	**−0.199**	**0.000**	**−0.500**	**0.000**	427

Significant correlations are marked in bold. VA, Viral abundance; PA, Prokaryotic abundance; N, number of analyzed data.

**Figure 7:**
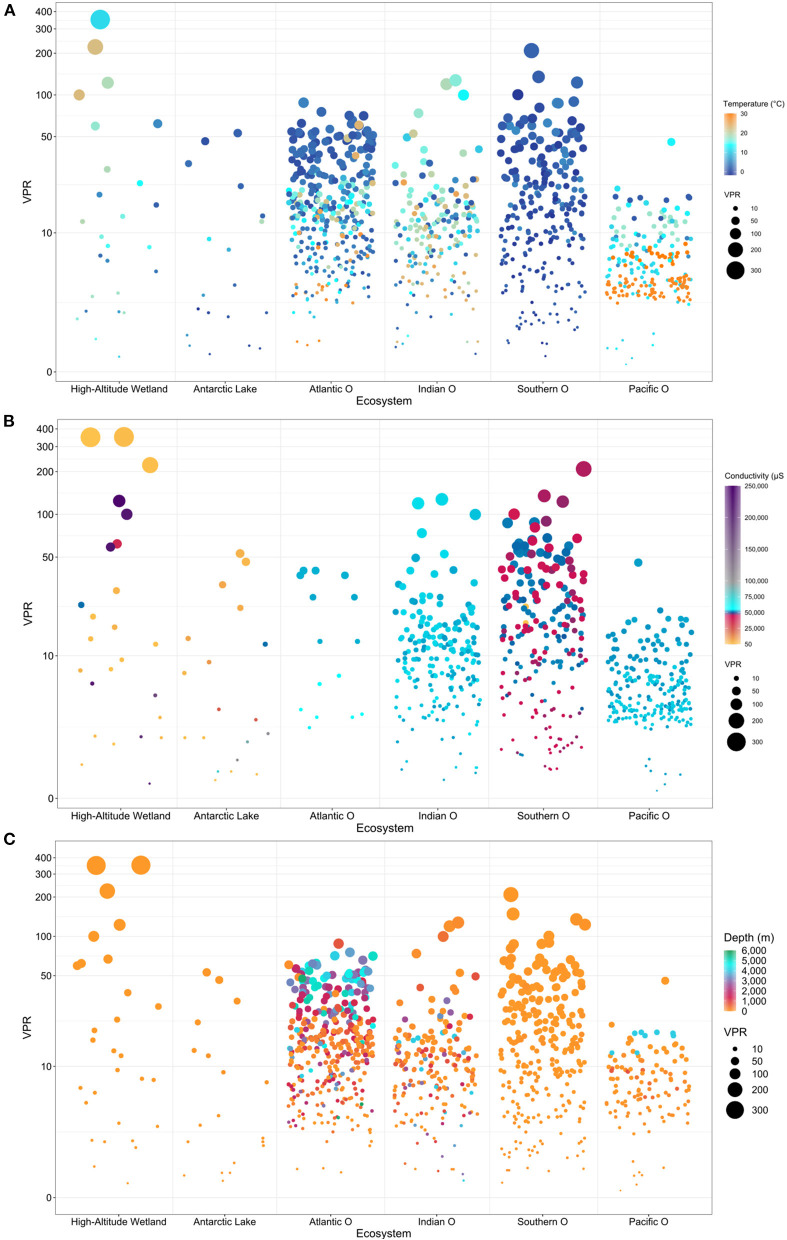
Bubble plots analysis **(A)** Temperature **(B)** Conductivity and **(C)** Depth, showing the relationship between environmental variables and VPR values potentially relevant in the different ecosystems analyzed.

Overall, bubble plots showed a high VPR variability associated with temperature, conductivity, and depth conditions (Figures 7A–C). Extreme environments, such as high-altitude wetlands and Antarctic lakes, presented the highest VPR variability compared with the oceanic ecosystems. However, when comparing the oceans, some trends were evidenced in the databases characterized by a higher VPR variability from the Southern, Indian, Atlantic compared with the Pacific Ocean. For seawater environments, the lowest and highest VPR values were recorded in the Pacific Ocean and Southern Ocean, respectively (Figures 7A–C). Most of the VPR data available was associated with the upper 1,000 m water depth for all marine systems and surface waters in terrestrial aquatic sites. Higher VPR values were concentrated at the surface compared with deep and cold waters in the Southern Ocean, whereas the opposite was observed in the Pacific Ocean Supplementary Figure S1). The highest VPR magnitudes were registered in high-altitude wetlands (Figure 7).

**Figure 8:**
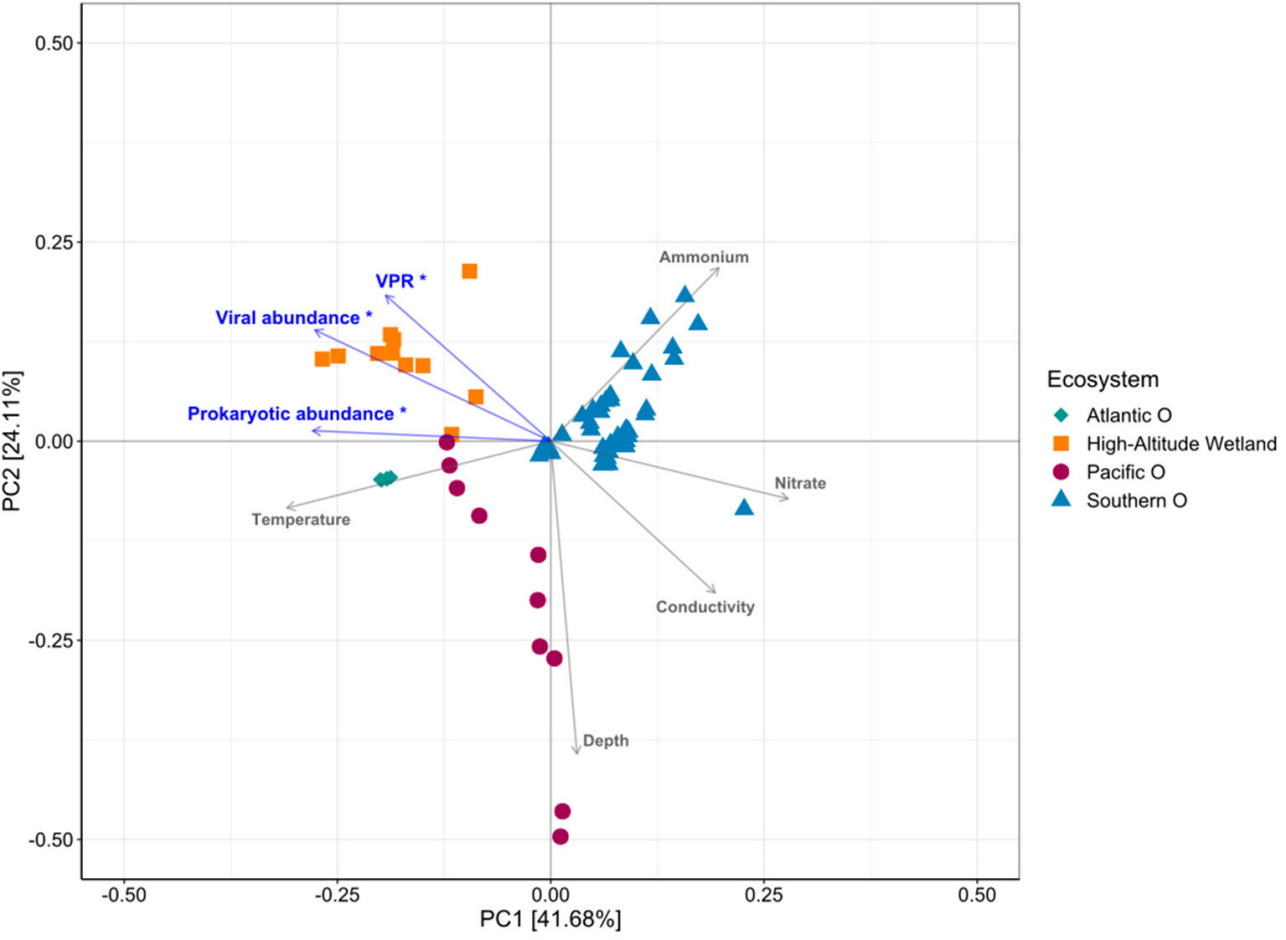
Principal component analysis showing the relationship between environmental variables and VPR values potentially relevant in the different ecosystems analyzed.

PCA analysis of environmental conditions, considering Atlantic, Pacific and Southern Ocean and high-altitude wetlands (Salar de Huasco and Salar de Uyuni), accounted for 41.68% in PC1 and 24.11% PC2 of the variability (Figure 8). The ordination showed that the aquatic systems were clearly differentiated on the PC1-axis, associated mainly with temperature, conductivity and nitrate; whereas depth and ammonium, on the PC2-axis. Viral and prokaryotic abundances and VPR were significantly related with the variability represented in PC1-axis, showing *r* = −0.89, −0.998 and −0.73, respectively (*P* ≤ 0.01) (Figure 8). The variability was mostly associated with the differentiation between high-altitude wetland compared with the other ecosystems.

## Discussion

In this study, we analyzed the virus-to-prokaryote ratio (VPR) values from two ponds of Salar de Huasco, including the latest available data for this ecosystem to explore the relationships between biological and physicochemical variables. In addition, to investigate general patterns in the relationship between VPR and environmental variables, metadata from other aquatic ecosystems from the Southern hemisphere was analyzed.

### VPR and environmental conditions in Salar de Huasco

Viral and prokaryotic abundance found in the PR and PV pond sites of Salar de Huasco were within the range found in previous studies, i.e., 7.78 × 10^5^ to 4.78 × 10^8^ VLP mL^−1^ for viral (VA) and 1.10 × 10^4^ to 1.83 × 10^7^ cells mL^−1^ for prokaryotes (PA) (Supplementary Table S1, Molina et al., 2018; Eissler et al., 2019, 2020). However, the VPR ranges (1.15–15.99) were lower than those reported for the study area (67–351), measured at similar aquatic sites but during the austral summer wet season in December–February (Eissler et al., 2019). Moreover, these VPR values were in the higher range found for most ecosystems (Wommack and Colwell, 2000; Parikka et al., 2016). Our results were consistent with the VPR ranges determined in other high-altitude wetlands such as Salar de Uyuni (0.51–122.5, Ramos-Barbero et al., 2019).

The aquatic areas of the ponds in Salar de Huasco are characterized by high variability in their environmental conditions associated with physicochemical changes such as conductivity, pH and nutrients (Figure 5). Salar de Huasco has been described as a hyper arid environment having different microhabitats with extreme differences in salinity (Risacher et al., 2003), which is a relevant environmental factor for microbial community composition (e.g., Dorador et al., 2020). Previous studies indicate that ponds are aquatic systems covering large surface areas of Salar de Huasco, which have a highly diverse and conspicuous microbial life contributing significantly with biogeochemical recycling processes (Aguilar et al., 2016; Hernández et al., 2016; Molina et al., 2020). During our study, the ponds analyzed presented a particular coloration (Figure 1) due to the presence of microbial mats with the predominance of bacterial communities, probably from the Roseobacter group in PR and Cyanobacteria in the case of PV, which have been previously identified in the Salar de Huasco (Dorador et al., 2008, 2013; Aguilar et al., 2016).

In addition, VA and PA cycled during the day in the PR pond, showed a maximum VPR value early in the morning compared to the afternoon, a trend supported in the PV pond comparing two sampling points (Figure 3). Extreme changes in the environmental conditions in this extreme ecosystem, including solar radiation, thermal and wind stress, have been found to shape microbial communities, influencing the nutrient and greenhouse gases reservoirs of the ponds of Salar de Huasco (Molina et al., 2020). In the current study, physicochemical (conductivity and pH) and biological (chlorophyll *a*) variables were correlated with VPR shifts (Supplementary Table S4). On the other hand, considering all the available data on this high-altitude wetland, significant correlations support the role of physicochemical conditions in shaping viral and prokaryotic abundance, including nutrients (Supplementary Table S5). The relationships found between viral dynamics and environmental conditions are consistent with other natural wetland types, such as tidal, mangrove, freshwater, forested, riparian and constructed wetlands, which are understudied land systems in comparison to marine systems (Jackson and Jackson, 2008), especially in the southern hemisphere. In the Salar de Huasco wetland, higher nutrient availability, mainly during the wet season, has been related with microbial activity and the different microhabitats, such as ponds, springs, and the lagoon (Eissler et al., 2019, 2020). Several authors have pointed out the importance of viruses in the cycling of nutrients, especially regarding the carbon cycle in aquatic ecosystems (e.g., Fuhrman, 1999; Wommack and Colwell, 2000; Weinbauer, 2004; Suttle, 2005, 2007; Breitbart et al., 2018). Recently, Gao et al. (2022), calculated that for wetlands from tundra ecosystems the contribution rate of carbon release by viral lysis of bacteria to the ecosystem's dissolved organic carbon ranged from 0.8 to 4.4, with an average of 2.6 ‰, representing a significant amount compared with marine ecosystems. In some wetlands, rewatering and water level conditions could change viral lysis rates which may vary the emissions of greenhouse gases in these systems as experimentally explored (Bonetti et al., 2021). So far, a high carbon release by viral lysis is expected in the high-altitude wetlands during the wet season and, particularly, at specific hours of the day, such as in the early morning periods, where greater dissolved organic carbon, nutrient and CH_4_ accumulation have been registered in diel cycling at pond sites (Molina et al., 2020).

### Comparison of viral dynamics in different ecosystems of the southern hemisphere

The ecosystems compared in this study presented large physicochemical and biological differences, in terms of salinity, nutrients (i.e., nitrate, nitrite, phosphate and silicate), temperature, dissolved oxygen, pH and chlorophyll (Supplementary Table S1; Figure 8), which can influence and regulate viral dynamics, virus-host interactions and, therefore, the VPR values in the ecosystems studied (Wommack and Colwell, 2000; Parikka et al., 2016). The high-altitude wetlands, located in the Andes where Chile, Bolivia and Argentina meet, are extreme environments presenting saline lakes fed by waters from the Andes mountains, characterized by high evaporation and low rainfall (Risacher et al., 2003; Risacher and Fritz, 2009). As mentioned before, Salar de Huasco is characterized by being a diverse and changing environment, with permanent brackish lagoons, ephemeral hypersaline or freshwater ponds (de la Fuente and Niño, 2010; Bull and Asenjo, 2013; Carrasco-Lagos et al., 2015). On the other hand, Salar de Uyuni (Ramos-Barbero et al., 2019; Figure 2, ID 2) stands out for being one of the world's largest and most hypersaline high-altitude wetlands (Rettig et al., 1980; Risacher and Fritz, 2009). In both wetlands, samples were collected from different habitats: ponds and lakes in the Salar de Huasco; ponds, pits, groundwater, and lagoon in the Salar de Uyuni (Ramos-Barbero et al., 2019). These extreme ecosystems exhibit the wider range of VPR magnitude compared with other environments in our database (Figure 6). This is consistent with Parikka et al. (2016) study showing that saline environments, including marine areas, presented a high range of VPR values, significantly higher compared to freshwater environments, groundwater and hot springs.

Particularly, despite high-altitude, wetlands presented vastly different environmental characteristics in comparison with the other ecosystems analyzed in our study. The viral activity measured using the VPR was not significantly different, except when compared with the Pacific Ocean VPR values (Table 2). The Pacific Ocean recorded the low average VPR values (7.65, 7.80 and 5.29, respectively) compared with the data registered in the Atlantic, Indian and Southern oceans (Figure 6; Table 1). The Pacific Ocean VPR variability could be associated with the temporal-spatial distribution of the data analyzed, such as, an oxygen minimum zone (OMZ) 135 miles west off Valparaíso, Chile (Chiang and Quiñones, 2007; Figure 2, ID 3), an upwelling system 18 and 40 miles west off Concepción, Chile (Eissler et al., 2010; Figure 2, ID 4) and surface waters in the Great Barrier Reef (Carreira et al., 2020; Figure 2, ID 6). The oxygen level related to depth in the Pacific Ocean also influences the distribution of VPR values, which decreased from the well-oxygenated surface part of the water column (from 12.56 on the surface to 4.3 at 100 m) (Chiang and Quiñones, 2007). Additionally, when hypoxic conditions were observed, a slight increase was detected (7.05 at 150 m) (Chiang and Quiñones, 2007). These variations in the VPR values in the oxic and anoxic layer were the result of different microbial assemblages that inhabit these strata (Chiang and Quiñones, 2007). Likewise, low VPR values observed in anoxic environments could be accounted for a reduction in viral production due to the decrease in the host communities or to a high loss of free-living viruses (Cassman et al., 2012). In contrast, despite the low VPR average observed off Concepción, the values ranged from 0.25 to 45.73, indicating a high viruses and prokaryotes dynamic and that during upwelling conditions VPR values were in fact higher (Eissler et al., 2010). In addition, Wommack and Colwell (2000) indicated that VPR values generally vary between 3 and 10, and that higher values are associated with highly productive environments (Wommack and Colwell, 2000; Weinbauer, 2004; Parikka et al., 2016), which has been also associated with upwelling environments of the Eastern Pacific (Kuznar et al., 2009; Letelier et al., 2009; Eissler et al., 2010). In two of these areas (Chiang and Quiñones, 2007; Carreira et al., 2020) nutrient availability was relatively low, especially in the Great Barrier Reef (Carreira et al., 2020). As an example, HPO${}_{4}^{2-}$ average was 0.09 ± 0.02 mmoL μL^−1^, therefore, this could have contributed to the overall lower VPR average and range observed.

Specifically, the VPR values of the Antarctic lakes showed significant differences from those of the Southern Ocean and high-altitude wetlands (Table 2). The McMurdo dry valleys in Antarctica (Lisle and Priscu, 2004; Figure 2, ID 16) are some of the most extreme ecosystems on the planet. Its frozen lakes are characterized by their perennial ice covers, consequently, they have poor nutrient input (McKay et al., 1985; Chinn, 1993). According to Lisle and Priscu (2004), this lack of nutrients (especially the shortage of phosphorus) would produce low viral activity and induce the lysogenic cycle. In addition, it would induce the formation of microbial aggregates around the available nutrients, thus allowing nutrients recycling (such as phosphate) within these aggregates, which would be promoted by viral lysis (Lisle and Priscu, 2004). On the other hand, the Southern Ocean presents a greater availability of nutrients that supports an increase in primary production (Tréguer and Jacques, 1992; Smith Jr et al., 2000), and, therefore, an increase in VPR values. Furthermore, Vaqué et al. (2017) and Sotomayor-Garcia et al. (2020) indicate that viral activity in Antarctic waters is more sensitive to temperature and prokaryotic abundance than to any other environmental factors. In contrast, high-altitude wetlands present a larger nutrient input, allowing a greater development of microbial and viral communities (Márquez-García et al., 2009; Haferburg et al., 2017; Eissler et al., 2019; Ramos-Barbero et al., 2019).

### Influence of environmental variables on the viral dynamics in the southern hemisphere ecosystems

Regardless of the methods used to count viruses and prokaryotes, 4 out of the 15 studies here use epifluorescence microscopy and the rest use flow cytometry (Supplementary Table S1). The tendencies of the relationships between VPR values and physicochemical variables found here were consistent with previous studies that analyzed a larger data set from a wider spatial-temporal scale (Wommack and Colwell, 2000; Parikka et al., 2016). First, as expected by solving the mathematical relationship between viral and prokaryotic abundance, using VPR (VPR = Viral abundance/Prokaryotic abundance) (Heldal and Bratbak, 1991; Parikka et al., 2016), VPR values were positively related to the viral abundance and negatively to the prokaryotic abundance, since there is an increase in the ratio when the viral abundance increases in relation to the prokaryotic abundance.

In the ecosystems studied, VPR was significantly positively correlated with chlorophyll *a* concentration (Table 3). These correlations were in agreement with Parikka et al. (2016) results in aquatic habitats and sediment-related ecosystems where VPR was positively correlated with VA, chlorophyll *a* and other microbial variables associated with infected cells (e.g., visibly infected cells and burst size). This positive correlation may suggest a viral infection on phytoplankton since chlorophyll *a* has been largely used as an indicator of biomass of photoautotrophic organisms and this relationship has numerous examples in literature as well as in seawater and in freshwater environments (Wommack and Colwell, 2000; Mojica and Brussaard, 2014; Zhong et al., 2014; Parikka et al., 2016; Flynn et al., 2022). Moreover, it has been suggested that this relationship indicates that viral activity regulates the structure of the phytoplankton community and the primary productivity of the marine ecosystems studied (Suttle, 1994; Boyer et al., 2009; Weitz et al., 2015).

Both temperature and conductivity in the ecosystems analyzed showed a significant negative relationship with VPR values, as has been reported by Parikka et al. (2016). In aquatic environments with high temperatures, the structural conformation of the viral lipid membranes and viral capsid proteins can be affected, which makes viruses and their host more susceptible to this environmental factor (Baudoux and Brussaard, 2005; Mojica and Brussaard, 2014). In contrast, low temperatures in polar environments do not inhibit viral activity and its potential to infect microbial communities (Anesio and Bellas, 2011). It has been observed that in Antarctic and Sub-Antarctic marine environments viral abundance is primarily related to temperature (Sotomayor-Garcia et al., 2020; Figure 2, ID 9-12). Furthermore, in areas surrounding the Antarctic peninsula (Vaqué et al., 2017; Figure 2, ID 13-15) viruses rather than grazers are more important agents of prokaryotic mortality. In addition, in our database, the relationship between VPR and temperature may be influenced by the data obtained from the areas studied in the Southern Ocean (Vaqué et al., 2017; Sotomayor-Garcia et al., 2020), where viral activity and prokaryotic production is more affected by temperature than by any other environmental factors. It is worth mentioning that viruses are resilient to low temperatures, since when weather conditions are unfavorable for prokaryotes growth, viruses change their lytic life strategy for a lysogenic one (Laybourn-Parry et al., 2007; Anesio and Bellas, 2011; Brum et al., 2017).

Regarding conductivity, the ecosystems examined in this study showed that albeit viral abundance had a non-significant relationship with conductivity, VPR values and PA had a significant negative and positive relationship, respectively (Table 3). Viruses and prokaryotic hosts are conditioned to their own tolerance to the habitat's salinity. In other words, high salinities can affect the permeability and structure of the prokaryotic cell wall, preventing the coupling of viruses to cells (Brown, 1964), thus reducing viral activity. Nonetheless, it has been observed that bacteriophages and archaeoviruses seem to be more tolerant to strong ionic changes, showing adaptations to saline environments (Kukkaro and Bamford, 2009). In high-altitude wetlands, viral groups belonging to Caudovirales, which mainly include viruses infecting prokaryotes, are predominant (Ramos-Barbero et al., 2019; Eissler et al., 2020) and probably highly adapted to extreme physicochemical conditions, such as significant variations in conductivity and temperatures, common to these ecosystems (Dorador et al., 2020).

In the ecosystems studied, the VPR values were also positively correlated with the concentrations of nitrate, ammonium and phosphate (Table 3). According to Weinbauer (2004), the availability of inorganic nutrients indirectly influences viral activity, through host metabolism. For example, it has been observed that adding phosphate to a microcosm experiment results in an increase of biomass, indirectly generating an increase in VPR values (Tuomi et al., 1995). Therefore, nitrate, nitrite, and phosphate assimilated by the prokaryotic communities for their metabolic processes, indirectly favors the proliferation of viruses (Breitbart et al., 2018). To support the aforementioned, an increase in VPR values has been related to the trophic gradient, finding higher values in nutrient-rich environments (Wommack and Colwell, 2000; Parikka et al., 2016). In marine environments, the high concentration of organic substrates and nutrients such as during upwelling seasons, favors the rapid development of prokaryotic communities, in turn, favoring a higher viral dynamic (Culley and Welschmeyer, 2002; Kuznar et al., 2009; Eissler et al., 2010). Conversely, it has been determined that the unavailability of nutrients results in a decrease in the VPR values in oligotrophic environments (Wommack and Colwell, 2000; Parikka et al., 2016). This has been observed in Antarctic lakes, where the lack of phosphate and other nutrients causes changes in the life strategy of viral communities, with the lysogenic cycle prevailing over the lytic cycle (Lisle and Priscu, 2004; Säwström et al., 2007), and for microorganisms inhabiting the deep-sea (Weinbauer et al., 2003; Williamson et al., 2008; Anderson et al., 2011). Consequently, among other responses, viral life strategy change is directly related to the host physiological capacity to increase their metabolism and adapt to the lack of nutrient availability (Mojica and Brussaard, 2014).

Despite the fact that our database is biased because of the predominance of marine environments, the incorporation of high-altitude wetlands and other extreme ecosystems support previously reported factors known to account for VA and VPR variability in the southern hemisphere. Conductivity and temperature gradients, as well as nutrients prevail as the main factors known to influence viral – host activity globally (Wells and Deming, 2006; Mojica and Brussaard, 2014; Finke et al., 2017).

## Conclusions

In Salar de Huasco, ponds with different characteristics have a variable microbial dynamic associated with viral and prokaryotic abundance that is reflected in the VPR values that variated with the time of the day and which is probably linked to the ponds' nutrient reservoir. The high-altitude wetlands, in comparison with other ecosystems with different conditions, showed a greater range of VPR values, as a consequence of the highly diverse microbial communities hosting viruses and the physicochemical gradients that structure aquatic life conditions, generating diverse niches such as permanent brackish lagoons, ephemeral hypersaline or freshwater ponds. Metadata analyses of southern ecosystems indicate that virus and prokaryotic dynamics were understudied in wetlands and in extreme ecosystems; thus, more studies to deeply characterize the role of viruses in these types of environments are required. In addition, the comparison that we have conducted provides information on key environmental factors shaping microbial community dynamics. In general, temperature, salinity and nutrients were identified to account for viral dynamics of the different ecosystems analyzed in this study. These findings confirm the relevance of the viral dynamics in the high-altitude wetlands potentially influencing nutrient and carbon dynamics which needs to be further explored in this extreme ecosystem.

## Data availability statement

The original contributions presented in the study are included in the article/Supplementary material, further inquiries can be directed to the corresponding authors.

## Author contributions

YE and VM designed the study. VM, YE, PC-P, CD, and MC-D'O organized the sampling and collected samples. AC-R, YE, and VM managed data mining and analyses, interpreted the results, and wrote the manuscript. PC-P, MC-D'O, and PA contributed to the data analyses. All authors contributed substantially to manuscript revisions and read and approved the final manuscript.

## Funding

This research was funded by FONDECYT, Grants Numbers 1181773 and 1211977.

## Conflict of interest

The authors declare that the research was conducted in the absence of any commercial or financial relationships that could be construed as a potential conflict of interest.

## Publisher's note

All claims expressed in this article are solely those of the authors and do not necessarily represent those of their affiliated organizations, or those of the publisher, the editors and the reviewers. Any product that may be evaluated in this article, or claim that may be made by its manufacturer, is not guaranteed or endorsed by the publisher.

## Supplementary material

Supplementary Table S1 All data used in this work to obtain and compare VPR values with physicochemical and biological variables from different ecosystems in the Southern hemisphere.

Supplementary Table S2 Normality test of biological and physicochemical variables for all ecosystems of Southern Hemisphere. Data that did not comply with the assumption of normality are presented in bold (*P* < 0.05). K-S, Kolmogórov-Smirnov Test; W, Shapiro-Wilk Test. P, probability and *N*, Number of analyzed data; VPR, viral to prokaryote ratio.

Supplementary Table S3 Values of viral (VA) and prokaryotic abundance (PA), VPR (viral to prokaryote ratio) and environmental parameters (Temp, water temperature; Cond, conductivity) for PR (“Poza Rosada”) and PV (“Poza Verde”) in Salar de Huasco. SD; standard deviation.

Supplementary Table S4 Spearman rank order correlations (r) matrix for Salar de Huasco variables. Significant correlations (*P* < 0.05) are marked in bold, *N* = 7. VA, viral abundance; PA, prokaryotic abundance, VPR, viral to prokaryote ratio, Temp, temperature, Cond, conductivity.

Supplementary Table S5 Spearman rank order correlations (r) matrix for Salar de Huasco variables using all data available (Molina et al., 2018; Eissler et al., 2019, 2020). Significant correlations (P < 0.05) are marked in bold. VA, viral abundance; PA, prokaryotic abundance; VPR, viral to prokaryote ratio; Temp, temperature; Cond, conductivity. VA, PA, VPR, Nitrite, Phosphate *N* = 21, Cond, pH, Nitrate *N* = 19, Ammonium, Dissolved Oxygen *N* = 14, Chlorophyll *a N* = 7.

Supplementary Table S6 Mean values of physicochemical parameters and nutrients in different ecosystems studied. Temp, temperature; Cond, conductivity; DO, Dissolve Oxygen; Ch-*a*, Chlorophyll *a*; NA, data not available.

Supplementary Figure S1 Bubble plots analysis showing VPR distribution with depth in the different ecosystems analyzed, **(A)** depth from 1–1,000 m, **(B)** depth from 1,000–6,000 m.
