# Supplementary material for: Virus-to-prokaryote ratio in the Salar de Huasco and different ecosystems of the Southern hemisphere and its relationship with physicochemical and biological parameters
Source: Front Microbiol. 2022 Aug 18;13:938066. doi: 10.3389/fmicb.2022.938066 (PMC9434117; doi:10.3389/fmicb.2022.938066)
Supplement: Supplementary Table S2 — Normality test of biological and physicochemical variables for all ecosystems of Southern Hemisphere. Data that did not comply with the assumption of normality are presented in bold (P < 0.05). K-S, Kolmogórov-Smirnov Test; W, Shapiro-Wilk Test. P, probability and N, Number of analyzed data; VPR, viral to prokaryote ratio. [file Table_2.docx]

**Supplementary Table S2**. Normality test of biological and physicochemical variables for all ecosystems of Southern Hemisphere. Data that did not comply with the assumption of normality are presented in bold (*P* < 0.05). K-S: Kolmogórov-Smirnov Test; W: Shapiro-Wilk Test. P: probability and N: Number of analyzed data. VPR: viral to prokaryote ratio.

|  | K-S *P* | Lilliefors *P* | W | P | N |
| --- | --- | --- | --- | --- | --- |
| Viral abundance | **p < 0.01** | **p < 0.01** | **0.215403** | **0.000000** | 1190 |
| Prokaryotic abundance | **p < 0.01** | **p < 0.01** | **0.213790** | **0.000000** | 1185 |
| VPR | **p < 0.01** | **p < 0.01** | **0.493866** | **0.000000** | 1177 |
| Temperature | **p < 0.01** | **p < 0.01** | **0.871291** | **0.000000** | 1053 |
| Salinity | **p < 0.01** | **p < 0.01** | **0.499236** | **0.000000** | 662 |
| Conductivity | **p < 0.01** | **p < 0.01** | **0.371646** | **0.000000** | 681 |
| Dissolved oxygen | **p < 0.01** | **p < 0.01** | **0.652649** | **0.000000** | 110 |
| pH | p > 0.20 | p > 0.20 | **0.906883** | **0.014375** | 29 |
| NO_3_^-^ | **p < 0.01** | **p < 0.01** | **0.562236** | **0.000000** | 327 |
| NO_2_^-^ | **p < 0.01** | **p < 0.01** | **0.515504** | **0.000000** | 100 |
| NH_4_^+^ | **p < 0.01** | **p < 0.01** | **0.688665** | **0.000000** | 101 |
| Silicate | **p < 0.01** | **p < 0.01** | **0.510463** | **0.000000** | 110 |
| Phosphate | **p < 0.01** | **p < 0.01** | **0.104669** | **0.000000** | 307 |
| Clorophyll *a* | **p < 0.01** | **p < 0.01** | **0.093510** | **0.000000** | 440 |

**Supplementary Table S3**. Values of viral (VA) and prokaryotic abundance (PA), VPR (viral to prokaryote ratio) and environmental parameters (Temp = water temperature, Cond= conductivity) for PR (“Poza Rosada”) and PV (“Poza Verde”) in Salar de Huasco. SD = standard deviation.

| **Sites** | **Time**  **(h)** | **VA**  **(VLP mL^-1^)** | **SD** | **PA**  **(cells mL^-1^)** | **SD** | **VPR** | **Temp**  **(°C)** | **Cond**  **(µS cm^-1^)** | **pH** | **NO_3_^-^  (µM)** | **SD** | **NO_2_^-^ (µM)** | **SD** | **Silicic acid**  **(µM)** | **SD** | **Phosphate (µM)** | **SD** |
| --- | --- | --- | --- | --- | --- | --- | --- | --- | --- | --- | --- | --- | --- | --- | --- | --- | --- |
| **PR** | 8:56 | 7.67x10^7^ | 4.63x10^6^ | 11.3x10^6^ | 9.93x10^5^ | 6.77 | 2.8 | NA | NA | 0.2 | 0.00 | 0.02 | 0.01 | 1053.5 | 242.5 | 3.21 | 0.00 |
|  | 11:30 | 5.73x10^7^ | 7.08x10^6^ | 7.32x10^6^ | 6.3E+04 | 7.83 | 14.6 | 3692 | 9.3 | 0.05 | 0.07 | 0.00 | 0.00 | 864 | 32.5 | 3.32 | 0.04 |
|  | 15:30 | 5.97x10^7^ | 2.16x10^6^ | 18.8x10^6^ | 9.21E+06 | 3.17 | 18 | NA | NA | 0.1 | 0.14 | 0.00 | 0.00 | 848 | 17.0 | 3.41 | 0.04 |
|  | 17:30 | 7.73x10^7^ | 7.34x10^6^ | 22.5x10^6^ | 4.22E+06 | 3.44 | 17.9 | 2176 | 9.08 | 0.1 | 0.00 | 0.03 | 0.04 | 880.5 | 37.5 | 3.45 | 0.06 |
|  | 6:30 | 9.01x10^7^ | 4.06x10^6^ | 5.64x10^6^ | 3.54x10^5^ | 15.99 | 1.7 | 3704 | 9.34 | 0.1 | 0.00 | 0.05 | 0.07 | 1012 | 145.7 | 3.34 | 0.04 |
|  |  |  |  |  |  |  |  |  |  |  |  |  |  |  |  |  |  |
| **PV** | 7:30 | 1.18x10^7^ | 1.46x10^6^ | 4.97x10^6^ | 1.53x10^5^ | 2.38 | 3.8 | 1547 | 8.74 | 0.4 | 0.14 | 0.02 | 0.03 | 491.5 | 17.7 | 0.71 | 0.02 |
|  | 11:30 | 0.94x10^7^ | 1.03x10^6^ | 8.17x10^6^ | 4.01x10^5^ | 1.15 | 15.3 | 1651 | 8.79 | 0.35 | 0.07 | 0.00 | 0.00 | 639 | 106.1 | 0.67 | 0.01 |

| **Sites** | **Time**  **(h)** | **Chlorophyll *a* (µg L^-1^)** | **SD** |
| --- | --- | --- | --- |
| **PR** | 8:56 | 0.39 | 0.05 |
|  | 11:30 | 0.32 | 0.01 |
|  | 15:30 | 1.15 | 0.02 |
|  | 17:30 | 1.06 | 0.04 |
|  | 6:30 | 0.86 | 0.02 |
|  |  |  |  |
| **PV** | 7:30 | 2.62 | 0.21 |
|  | 11:30 | 2.82 | 0.21 |

NA: Data not available

**Supplementary Table S4:** Spearman rank order correlations (r) matrix for Salar de Huasco variables (September 2019). Significant correlations (P < 0.05) are marked in bold, N = 7. VA: viral abundance; PA: prokaryotic abundance, VPR: viral to prokaryote ratio, Temp: temperature, Cond: conductivity.

|  | VA | PA | VPR | Temp | Cond | pH | Nitrate | Nitrite | Silicic acid | Phosphate | Chlorophyll *a* |
| --- | --- | --- | --- | --- | --- | --- | --- | --- | --- | --- | --- |
| VA | 1 | 0.286 | 0.750 | -0.286 | 0.800 | 0.800 | -0.482 | **0.767** | **0.821** | 0.750 | -0.536 |
| PA | 0.286 | 1 | -0.107 | 0.679 | 0.100 | 0.100 | -0.297 | -0.112 | 0.321 | 0.571 | -0.036 |
| VPR | 0.750 | -0.107 | 1 | -0.536 | **0.900** | **0.900** | -0.704 | 0.468 | **0.786** | 0.464 | **-0.893** |
| Temp | -0.286 | 0.679 | -0.536 | 1 | -0.300 | -0.300 | -0.185 | -0.543 | -0.429 | 0.357 | 0.393 |
| Cond | 0.800 | 0.100 | **0.900** | -0.300 | 1 | 1 | -0.821 | 0.410 | **0.900** | 0.600 | -0.800 |
| pH | 0.800 | 0.100 | **0.900** | -0.300 | 1 | 1 | -0.821 | 0.410 | **0.900** | 0.600 | -0.800 |
| Nitrate | -0.482 | -0.297 | -0.704 | -0.185 | -0.821 | -0.821 | 1 | 0.039 | -0.445 | -0.704 | 0.704 |
| Nitrite | **0.767** | -0.112 | 0.468 | -0.543 | 0.410 | 0.410 | 0.039 | 1 | 0.524 | 0.356 | -0.187 |
| Silicic acid | **0.821** | 0.321 | 0.786 | -0.429 | **0.900** | **0.900** | -0.445 | 0.524 | 1 | 0.429 | -0.750 |
| Phosphate | 0.750 | 0.571 | 0.464 | 0.357 | 0.600 | 0.600 | -0.704 | 0.356 | 0.429 | 1 | -0.357 |
| Chlorophyll *a* | -0.536 | -0.036 | **-0.893** | 0.393 | -0.800 | -0.800 | 0.704 | -0.187 | -0.750 | -0.357 | 1 |

**Supplementary Table S5:** Spearman rank order correlations (r) matrix for Salar de Huasco variables using all data available (Molina et al., 2018; Eissler et al., 2019, 2020, this study). Significant correlations (P < 0.05) are marked in bold. VA: viral abundance; PA: prokaryotic abundance, VPR: viral to prokaryote ratio, Temp: temperature, Cond: conductivity. VA, PA, VPR, Nitrite, Phosphate N = 21, Cond, pH, Nitrate N = 19, Ammonium, Dissolved Oxygen N = 14, Chlorophyll *a* N = 7.

|  | VA | PA | VPR | Temp | Cond | pH | Nitrate | Nitrite | Silicid acid | Phosphate | Ammonium | Dissolved Oxygen | Chlorophyll *a* |
| --- | --- | --- | --- | --- | --- | --- | --- | --- | --- | --- | --- | --- | --- |
| VA | 1 | **0.571** | **0.451** | -0.170 | **0.605** | **0.499** | -0.401 | 0.114 | **0.555** | 0.358 | -0.044 | -0.130 | -0.536 |
| PA | **0.571** | 1 | -0.390 | -0.142 | **0.635** | 0.309 | **-0.752** | -0.270 | 0.473 | 0.387 | -0.011 | -0.433 | -0.036 |
| VPR | **0.451** | -0.390 | 1 | 0.031 | 0.039 | 0.113 | 0.366 | 0.400 | 0.154 | 0.148 | -0.108 | 0.332 | **-0.893** |
| Temp | -0.170 | -0.142 | 0.031 | 1 | -0.448 | 0.142 | 0.188 | -0.249 | -0.451 | -0.278 | **-0.583** | 0.310 | 0.393 |
| Cond | **0.605** | **0.635** | 0.039 | -0.448 | 1 | 0.302 | **-0.732** | -0.235 | **0.727** | **0.598** | 0.319 | -0.165 | -0.800 |
| pH | **0.499** | 0.309 | 0.113 | 0.142 | 0.302 | 1 | **-0.643** | **-0.495** | 0.405 | -0.208 | -0.204 | 0.322 | -0.800 |
| Nitrate | -0.401 | **-0.752** | 0.366 | 0.188 | **-0.732** | **-0.643** | 1 | **0.644** | -0.596 | -0.315 | 0.035 | 0.168 | 0.704 |
| Nitrite | 0.114 | -0.270 | 0.400 | -0.249 | -0.235 | **-0.495** | **0.644** | 1 | 0.045 | 0.216 | 0.390 | -0.521 | -0.187 |
| Silicid acid | **0.555** | 0.473 | 0.154 | -0.451 | **0.727** | 0.405 | -0.596 | 0.045 | 1 | **0.742** | 0.029 | -0.600 | -0.750 |
| Phosphate | 0.358 | 0.387 | 0.148 | -0.278 | **0.598** | -0.208 | -0.315 | 0.216 | **0.742** | 1 | 0.411 | -0.402 | -0.357 |
| Ammonium | -0.044 | -0.011 | -0.108 | **-0.583** | 0.319 | -0.204 | 0.035 | 0.390 | 0.029 | 0.411 | 1 | -0.132 |  |
| Dissolved Oxygen | -0.130 | -0.433 | 0.332 | 0.310 | -0.165 | 0.322 | 0.168 | -0.521 | -0.600 | -0.402 | -0.132 | 1 |  |
| Chlorophyll *a* | -0.536 | -0.036 | **-0.893** | 0.393 | -0.800 | -0.800 | 0.704 | -0.187 | -0.750 | -0.357 |  |  | 1 |

**Supplementary Table S6**. Mean values of physicochemical parameters and nutrients in different ecosystems studied. Temp: temperature; Cond: conductivity; DO: Dissolve Oxygen; Ch-*a*: Chlorophyll *a*. NA: data not available.

| **Ecosystem** | **Temp** | **Salinity** | **Cond** | **DO** | **pH** | **NO_3_^-^** | **NO_2_^-^** | **NH_4_^+^** | **Silicate** | **Phosphate** | **Ch *a*** |
| --- | --- | --- | --- | --- | --- | --- | --- | --- | --- | --- | --- |
|  | (°C) | PSU | (µS cm^-1^) | (mg L^-1^) |  | (µM) | (µM) | (µM) | (µM) | (µM) | (μg L^-1^) |
| Salar de Huasco | 14.22 | 2.96 | 4838 | 7.39 | 8.46 | 5.69 | 0.13 | 0.60 | 766,42 | 18.20 | 0.81 |
| Salar de Uyuni | 12.20 | 31.57 | 216,285 | NA | 6.59 | NA | NA | NA | NA | NA | NA |
| Bajo O´Higgins Seamount, Valparaíso | NA | 34.37 | NA | NA | NA | NA | NA | NA | NA | NA | 0.01 |
| Coastal upwelling system, Concepción | 11.96 | 34.49 | 52,365 | NA | NA | 14.88 | 0.82 | 0.61 | 17.34 | 2.10 | 8.12 |
| Thermohaline circulation, South Pacific | 1.23 | 34.72 | 52,681 | NA | NA | NA | NA | NA | NA | NA | NA |
| Great Barrier Reef, Australia | 29.25 | 35.48 | 53,704 | NA | NA | 0.14 | NA | NA | NA | 0.07 | 0.32 |
| Sub-Antarctic Zone | 12.20 | 34.73 | 52,700 | NA | NA | 6.53 | NA | NA | 0.9 | 0.57 | NA |
| Polar Frontal Zone | 5.38 | 33.80 | 51,443 | NA | NA | 24.68 | NA | NA | 0.68 | 1.58 | NA |
| North of South Orkney | 0.33 | 33.94 | 51,632 | NA | NA | 27.29 | 0.23 | 2.30 | 48.10 | 1.98 | 1.74 |
| South of South Orkney | -1.11 | 33.59 | 51,153 | NA | NA | 27.63 | 0.17 | 1.9 | 47.45 | 2.13 | 0.39 |
| **Ecosystem** | **Temp** | **Salinity** | **Cond** | **OD** | **pH** | **NO_3_^-^** | **NO_2_^-^** | **NH_4_^+^** | **Silicate** | **Phosphate** | **Ch *a*** |
|  | (°C) | PSU | (µS cm^-1^) | (mg L^-1^) |  | (µM) | (µM) | (µM) | (µM) | (µM) | (μg L^-1^) |
| North of South Georgia | 4.55 | 33.76 | 51,381 | NA | NA | 22.02 | 0.29 | 1.85 | 2.06 | 1.26 | 6.43 |
| West of Anvers | 1.50 | 33.43 | 50,936 | NA | NA | 18.71 | 0.19 | 3.08 | 49.69 | 1.79 | 3.88 |
| Bransfield Strait | 1.30 | 29.56 | 45,632 | NA | NA | NA | NA | NA | NA | NA | NA |
| Bellingshausen Sea | 1.26 | 28.96 | 44,806 | NA | NA | NA | NA | NA | NA | NA | NA |
| Weddell Sea | -0.50 | 27.90 | 43,358 | NA | NA | NA | NA | NA | NA | NA | NA |
| McMurdo Dry Valleys, Antarctica | 3.77 | 18.84 | 27,219 | 23.56 | NA | NA | NA | NA | NA | NA | 2.67 |
| Thermohaline circulation, South Atlantic | 2.67 | 34.88 | 52,908 | NA | NA | NA | NA | NA | NA | NA | NA |
| Atlantic Ocean | 7.95 | 36.95 | 55,680 | NA | NA | 0.57 | 0.04 | 0.02 | 0.69 | NA | NA |
| Indian Ocean | 14.10 | 35.26 | 53,412 | NA | NA | NA | NA | NA | NA | NA | NA |
