# Supplementary material for: Virus-to-prokaryote ratio in the Salar de Huasco and different ecosystems of the Southern hemisphere and its relationship with physicochemical and biological parameters
Source: Front Microbiol. 2022 Aug 18;13:938066. doi: 10.3389/fmicb.2022.938066 (PMC9434117; doi:10.3389/fmicb.2022.938066)
Supplement: Supplementary Figure S1 — Bubble plots analysis showing VPR distribution with depth in the different ecosystems analyzed, (A) depth from 1–1,000 m, (B) depth from 1,000–6,000 m. [file Data_Sheet_1.docx]

**Supplementary Figures**


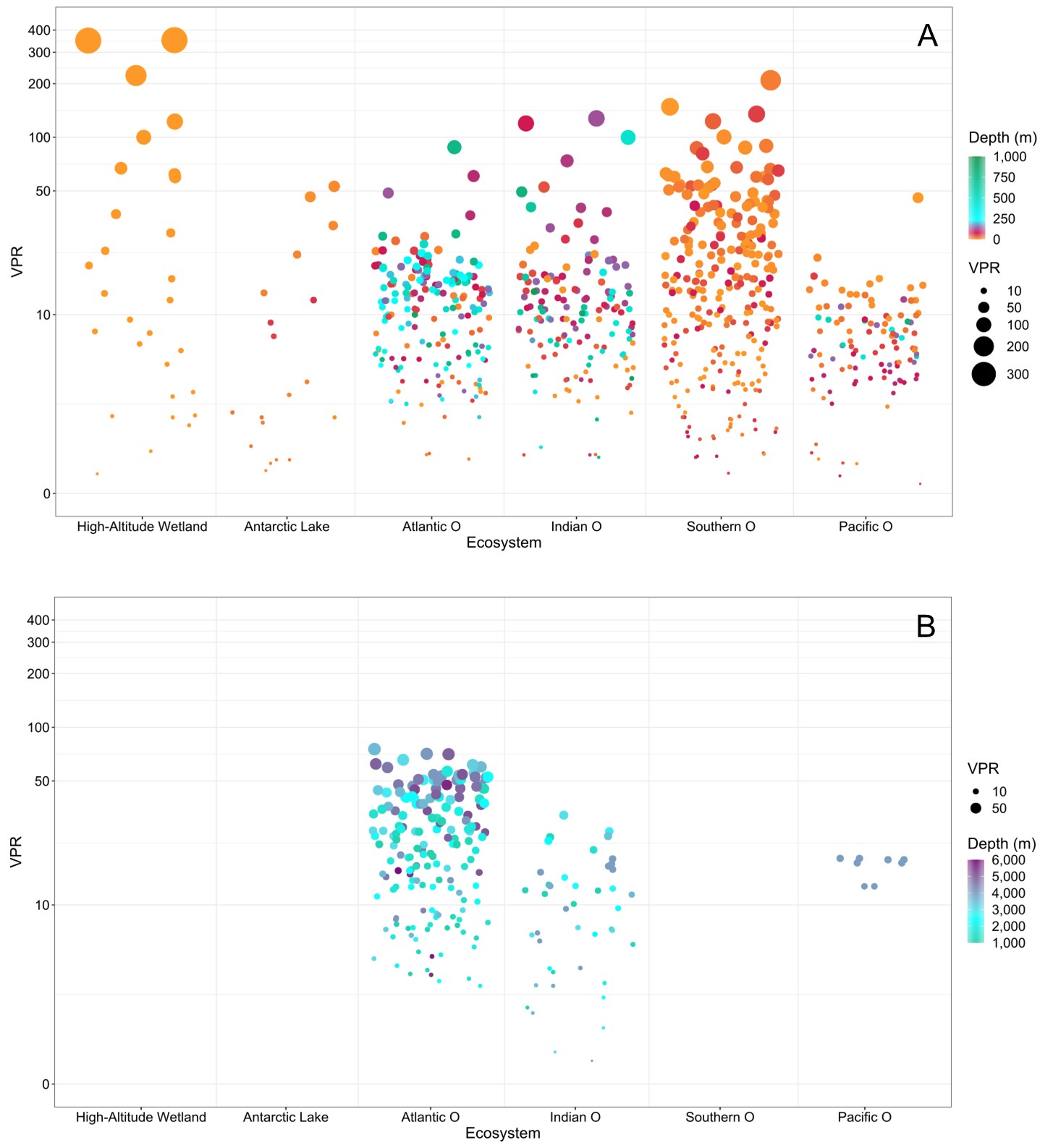


**Supplementary Figure S1**: Bubble plots analysis showing VPR distribution with depth in the different ecosystems analyzed, A) depth from 1-1000 m, B) depth from 1000-6000 m.
